# The middle-income trap in Central and Eastern Europe in the 2010s: institutions and divergent growth models

**DOI:** 10.1057/s41295-021-00264-3

**Published:** 2021-11-02

**Authors:** Dóra Győrffy

**Affiliations:** Institute of Economic and Public Policy, Corvinus University of Budapest, Fővám tér 8, 1093 Budapest, Hungary

**Keywords:** Central and Eastern Europe, Economic convergence, Estonia, Growth models, Hungary, Middle-income trap

## Abstract

The paper evaluates the convergence paths of Central and Eastern European member states of the EU during the 2010s, when the main task for these countries was avoiding the middle-income trap—when wages are not so low anymore to compete with less developed countries, while innovation is not developed enough yet to compete with developed countries. Using various statistical indicators, the paper shows that while most countries in the region have been on a convergence path during the decade under analysis, not all succeeded in avoiding the trap. While some countries successfully implemented policies to step on the path of productivity- and innovation-led growth (Czechia, Slovenia, Estonia, and Lithuania), in several other states, growth was supported mainly by low costs and loose monetary conditions including significant transfers from the EU. The comparative analysis of Estonia and Hungary illustrates the different growth models and shows how the institutional system plays a key role in exiting the trap.

## Introduction

The COVID-19 pandemic and the ensuing economic crisis ended a decade-long period of growth around the world and the Central and Eastern European (CEE) region, which warrants taking stock. This study provides an overview of the developmental paths of the 11 Central and Eastern European Member States during the 2010s: Bulgaria, Czechia, Estonia, Croatia, Poland, Latvia, Lithuania, Hungary, Romania, Slovakia and Slovenia (EU-11). The paper aims to answer three closely related questions. 1. What were the main characteristics of convergence in the region during the 2010s? 2. Have the EU-11 succeeded in overcoming the problem of the so-called middle-income trap (MIT)? If so, what strategies have been used? 3. What role did the institutional system play in this process?

To answer the three questions, first I present some stylized facts about the convergence performance of the EU-11. Then, to explain the differences in performance, I will provide a theoretical summary of the problem of the MIT, discuss different growth models and the role of institutions in the process. “Divergent growth models in the EU‑11” section examines the experiences of the EU-11 over the past decade based on the theory of MIT. It is shown that there are two distinct growth models emerging in the region—a high-road model that prioritises institution building, improvements in knowledge, quality and productivity, and a low-road model, which focuses on increasing demand and cost advantage. To illustrate the differences between the two models and the role of institutions in choosing among growth models, I compare the cases of Hungary and Estonia. The main finding of the paper is that high-quality institutions are necessary for overcoming the MIT, but given the challenges of institutional change, the MIT has not been overcome everywhere in the region thus growth based on low costs and favourable financial conditions persist even though it cannot be considered sustainable.

## Convergence performance of the EU-11 during the 2010s

**Fig. 1:**
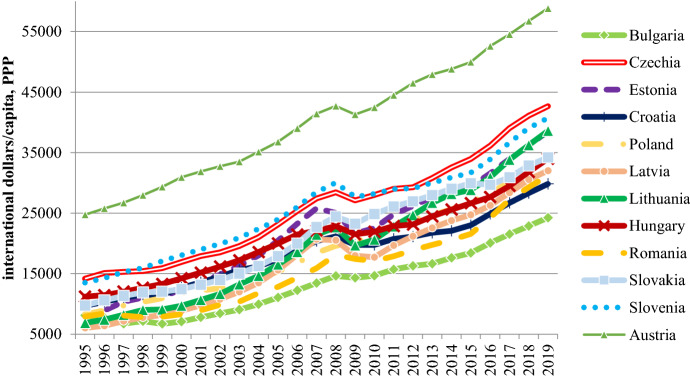
GDP/capita in the EU-11 and Austria, 1995–2019 (current prices, international dollars/capita). Data: IMF World Economic Outlook database

At the time of post-Communist transition, the promise of freedom and welfare intertwined—most citizens equated democracy and capitalism with the standard of living in Western countries such as Austria. Accession to the European Union (EU) also fuelled these expectations, as the free movement of goods, capital, labour, services and technology promote convergence (Sala-i-Martin 1996). Given their lower initial level of income EU-11 countries were expected to grow faster than old member states (β-convergence), which helps to reduce the dispersion of per capita income within the EU (σ-convergence). Nevertheless, 30 years after the transition, none of the post-Communist countries have achieved even the average level of development of the EU, let alone that of Austria. This is illustrated by Fig. 1, which shows that although there has been a significant increase in GDP between 1995 and 2019, Austria also developed rapidly and thus the distance barely decreased.

**Fig. 2:**
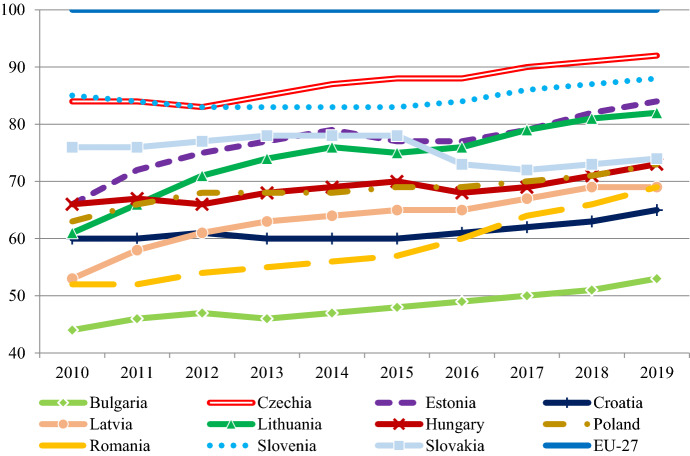
Convergence in the EU-11 2010–2019 (GDP/capita PPS). Data: Eurostat (data code: TEC00114)

While catching up with Austria has proven to be elusive, convergence has still taken place. Figure 2 shows changes in GDP per capita adjusted for purchasing power standard (PPS) between 2010 and 2019—after the global financial crisis and before the COVID-19 pandemic. All countries except Slovakia moved closer to the EU average (σ-convergence), with Czechia being the closest to catch up to the EU-27 average.

The concept of β-convergence predicts that the larger the initial difference from the EU average, the steeper a country's growth curve can be. Thus, to evaluate convergence performance during the 2010s, the initial level of development needs to be considered. Table 1 ranks countries based on their 2019 GDP/capita levels. The ‘Convergence’ indicator shows the percentage of the gap that was closed relative to the EU-27 average between 2010 and 2019.^1^ For example, Lithuania stood at 61% of the EU-27 average in 2010, which means that there was a 39 percentage points gap to close before catching up. By 2019, it had reached 82% of the EU-27 average, thus the gap was reduced to 18 percentage point. The 21-percentage point difference is 53,8% of the road to catch up.

**Table 1:** Convergence performance in the EU-11, 2010–2019

	GDP PPS 2010 (EU27 = 100)	GDP PPS 2019 (EU27 = 100)	ΔGDP (% pont)	Convergence (%)	Real GDP/capita 2019 (euro)	Δpopulation (%)
Czech Republic	84	92	8	50	18,330	1.6
Slovenia	85	88	3	20	20,700	1.8
Estonia	66	84	18	52,9	15,760	–0.5
Lithuania	61	82	21	53,8	14,010	–13
Slovakia	76	74	–2	–8,3	15,860	0.4
Poland	63	73	10	27	13,020	–1.3
Hungary	66	73	7	20,5	13,270	–2.7
Latvia	53	69	16	34	12,510	–11
Romania	52	69	17	35,4	9110	–6
Croatia	60	65	5	12,5	12,450	–5.2
Bulgaria	44	53	9	16,1	6840	–6.4

Data: GDP/capita PPS: Eurostat (code: TEC00114), convergence: own calculation, real GDP/capita: Eurostat (code: SDG_08_10), population: https://www.worldometers.info/population/europe

There is a significant heterogeneity in the convergence performance of the EU-11. Two Baltic States (Lithuania and Estonia) and Czechia performed the best as they closed over 50% of their development gap during the past decade. Romania and Latvia also performed well and closed around third of the gap. Poland, Hungary and Slovenia are in the middle with performances between 20 and 30%, while Bulgaria and Croatia are poor performers with 16% and 12.5% respectively. While Slovakia was a success story in the 2000s, in the 2010s its performance deteriorated compared to the region, and it is the sole country that have a lower level of development relative to the EU-27 average than in 2010.

When assessing the performance in GDP per capita, it is also worth considering changes in the population. As can be seen from the table, Slovakia is one of only three countries (alongside Czechia and Slovenia) that have been able to increase their population over the past decade, while other countries (especially Lithuania and Latvia) have caught up on a GDP/capita basis with rapidly declining populations.

What explains this heterogeneity? To address this question, first I will review the literature on the MIT and the most important lessons for the region.

## The limits to FDI-led growth, the MIT, and institutions

Due to the lack of capital and technology, which characterized post-Communist countries at the time of transition, they relied on foreign direct investment (FDI) and export-oriented growth to achieve convergence. Hungary and Estonia played a leading role in stepping on this path, but after the failure of the ideas of national capitalism, Czechia, Slovakia, Poland, and Slovenia also opted for this strategy (Csaba, 2005: 112–113). This model was also encouraged by the process of accession to the European Union—privatization and facilitation of FDI inflows were important aspects of evaluation (Medve-Bálint, 2014: 42). No alternative had emerged.

By the end of the first decade of the 2000s, a distinct model of capitalism developed in the post-Communist countries that does not fit into the categories used to describe the old member states of the EU (Farkas, 2016: 173). CEE countries shared three common features: shortage of capital and management skills, weak civil society, and the influence of the EU and other international organizations (Farkas, 2016: 175). Advanced technology is present in FDI-dominated sectors, where investors benefit from relatively cheap but skilled labor, access to new markets and the EU regulatory environment (Jirasavetakul and Rahman, 2018: 5–6). However, the productivity of the domestic economy lags foreign-dominated sectors, which means that a dual economic structure became characteristic to varying degrees everywhere in the region (Farkas, 2016: 211). Divergence in the region can be found primarily in the ratio of state redistribution, which ranges from 35 to 50% of GDP (see Fig. 4 in the next section).

By the 2010s, however, it had become increasingly clear that the post-transition growth model was becoming obsolete (Galgóczy and Drahokoupil 2017). The first major sign of this problem was during the global financial crisis, which started in 2008—compared to the previous periods, FDI flows decreased significantly across the world. As multinationals are increasingly looking for skilled labor, the lack of investment in education and the loss of population in several countries make it difficult to attract new FDI. A specialization in labor-intensive, lower value-added activities in global value chains can be an additional drawback of FDI-based growth. Finally, Industry 4.0 innovations, such as the rise of robots, 3D printing, artificial intelligence and digitalization are likely to further reduce efficiency-seeking FDI flows, which are primarily attracted by cheap labor.

The limitations of growth based on foreign technology and cheap labor are addressed in the concept of MIT. The phenomenon was first described by Garrett (2004), who pointed out that in the 1980s and 1990s, per capita income growth in middle-income countries was barely half of the growth in high-income countries and one-eighth of that of low-income countries. The reason is that their wages are no longer so low that they have a competitive advantage, while their innovation performance is not yet strong enough to compete with developed countries. The concept of MIT was first used by Gill and Kharas (2007), who considered it suitable for explaining the development problems of East Asia, the Middle East, and Latin America. They pointed to two forms of the trap: forcing export-oriented growth based on cheap labor, even after the wage advantage was lost, and the hasty leap towards a knowledge economy with weak universities, limited human capital, and waste of budgetary resources due to corruption and rule of law problems (Gill and Kharas 2015: 7).

Initially, the MIT concept was applied only below a certain level of development. However, due to widespread popularity of the concept in scholarly and media circles, it eventually obtained a much broader meaning. Nowadays, it basically refers to the lack of convergence to target countries, and it is this meaning, which has been widely applied to the CEE region (Pruchnik and Zowczak 2017: 2).

Recognizing the limitations of growth based on cheap labor and the importance of innovation is an important message of the MIT concept for economic policy. This idea is not new, of course, since Porter (1990) had already demonstrated the stages of competitive development long before: factor-oriented development is followed by the investment-driven phase and then innovation becomes decisive. Different factors contribute to competitiveness at different levels of development. One of the most recent approaches to the competitiveness of national economies is provided by Aiginger (2018), who distinguishes two types of competitiveness. Low-road competitiveness is focused on low costs (wages and taxes), subsidies for FDI inflow and aims for jobs and GDP growth. High-road competitiveness focuses on quality, sophisticated products, high level of productivity, which rely on innovation, quality education and a favorable business environment. The aim is improving the quality of life. Table 2 provides an overview about the two models of competitiveness.

**Table 2:** Low-road vs high-road approaches to competitiveness

	Low-road strategy	High-road strategy
Competitive advantage	Low costs (wages, energy, taxes)	Quality, sophisticated products, productivity
Growth drivers	Subsidies, dual labor markets, inward FDI	Innovation, education, universities, clusters
Ambitions	Cost advantage, flexible labor	Social empowerment, ecological excellence, trust
Instruments	Import taxes, protectionism, devaluation (external, internal)	Business environment, entrepreneurship, dialogue
Objectives	Catching up in GDP per capita, employment	Beyond GDP goods (income, social, ecological pillars)

*Source*: Aiginger (2018): 106

Based on the distinction between low-road and high-road competitiveness, the problem of MIT can be understood as a failure to convert from a growth model focusing on low costs to a model, which prioritizes high quality and innovation. Numerous economic policy recommendations have been made to address the MIT problem. Gill and Kharas (2015) provides a summary, which includes technological convergence, innovation, improving the skills of the labor force, livable cities and strengthening institutions. In the context of the latter, the authors highlight democratization, the fight against corruption, strengthening social cohesion, reducing inequality, and increasing government efficiency. In addition, experience has shown that demographic processes, entrepreneurship, and regional integration are also important. However, despite the knowledge about this long list of factors, the MIT persists, thus the question remains: why some countries can solve the problems, while others remain trapped?

Lately, the deeper, social, and institutional causes of the trap came into focus. Dollar (2016) points out that although there is a strong correlation between institutional quality and the level of development, the volatility of growth is far greater than that of institutions. Consequently, the question is not the level of institutional quality, but rather the quality of institutions relative to the level of development. He argues that rapid development becomes possible when a country succeeds in establishing higher quality institutions than could be expected from its income level. This also implies that institutional quality should be constantly improved to achieve steady convergence to target countries.

The focus on institutions in exiting the MIT is well in line with the institutional turn in economics since the 1990s. According to Douglass North (1990: 6) “the major role of institutions in a society is to reduce uncertainty by establishing a stable (but not necessarily efficient) structure to human interaction.” This insight did not remain at a theoretical level but has been emphasized in policymaking as well. In 2002 the World Bank identified three major channels, which support market functioning: providing information on market conditions, goods, and participation; defining and enforcing property rights and contracts; and increase or decrease in competition on a certain market (World Bank 2002: 8). However, a state, which is capable of enforcing contracts, can also abuse its power and jeopardize property rights. Thus there is a need for ‘restraint on government tyranny’, which means the rule of law (Tamahana 2004: 115). Historically it has three core elements: the government must respect the law; the laws are public, prospective, general and applied equally; and an independent judiciary is responsible for enforcing the law (Tamahana 2004: 114–126). The rule of law is not the only type of constraint on the state. The ratio of state redistribution also influences the autonomy of the state. A larger state provides more opportunities for corruption, abuse of power than a smaller state, although in other cases large governments provide high-quality services, which increase social cohesion and generate trust. For this reason, it is important to analyze the size of government and the quality of state institutions together (Győrffy 2018: 199). In an environment with limited government and high-quality institutions, where contracts are enforced and property is secure, entrepreneurs can flourish and drive innovation, which improves productivity and leads to economic growth (Ács et al. 2018: 505).

In their survey Haggard et al. (2008) find robust evidence for the link between long-term economic development and security of property rights ensured by the legal system. However, they warn against viewing rule of law as a technology and underline the complexity of institutions, which “rest upon deep coalitions of consenting interests” (221). This insight explains why an import of formal institutions often fails and past institutional practices are persistent. North (2005: 62) defines this phenomenon as path dependence, which “will occur because the direction of the incremental institutional change will be broadly consistent with the existing institutional matrix … and will be governed by the kinds of knowledge and skills that the entrepreneurs and members of organization have invested in.”

Research on the MIT is consistent with the insights of institutional economics on the difficulties of institutional change. Doner and Schneider (2016) underline that the actors, who benefit from the low-cost model, are not necessarily interested in institutional changes. Furthermore, establishing a high-quality education and R&D system requires much more complex policies than giving tax relief for multinational corporations attracted by low wages. The dual economy also creates significant inequalities in the system, which make it difficult to cooperate for a common purpose. The beneficiaries of the system, including employers of the low-wage workers and the rent-seeking policymakers do not have any interest in facilitating the changes. In such an environment, the developmental trap and the institutional trap reinforce each other.

A low-quality institutional environment however does not necessarily imply low level of growth—at least not in the short-term. As Baccaro and Pontusson (2016) show there are several possible models of growth even in the absence of productivity growth. While they apply their framework to developed countries, the various models are also open to countries dealing with the MIT. Wages can be kept artificially low, which sustains export competitiveness such as in Germany, or an increase in wage-share or credit can finance consumption such as in the UK. Sweden has the most balanced model focusing on knowledge-intensive and high value-added sectors that ensure robust growth for both exports and consumption. The EU-11 countries have some additional opportunities for growth. The influx of transfers from the EU can also lead to investments and increasing demand. In the short term, non-eurozone countries can improve competitiveness by devaluation, which makes domestic products cheaper abroad thus provides a cost advantage for producers. In the longer term, however, the link between financial conditions and growth is much more nuanced. Readily available resources may finance investments, which are profitable only during the expansive and peak stages of the business cycle. The emergence of various asset price bubbles on the stock or real estate market due to cheap funding can also be dangerous and lead to a crisis, as they collapse when monetary conditions tighten. An undervalued exchange rate is not necessarily a lasting solution to competitiveness either, as continued devaluation can have an inflationary effect, while it reduces the incentives for companies to increase productivity and innovate.

Overall, we can expect high-road, quality-growth in countries where we can see a steady improvement in institutional quality. In the absence of such improvement, growth is still possible based on costs or lax monetary conditions. If neither institutions, nor lax monetary conditions are present, we can expect economic stagnation. In the following section indicators of the various growth models are analyzed in the EU-11.

## Divergent growth models in the EU-11

### Institutional quality and entrepreneurship

**Fig. 3:**
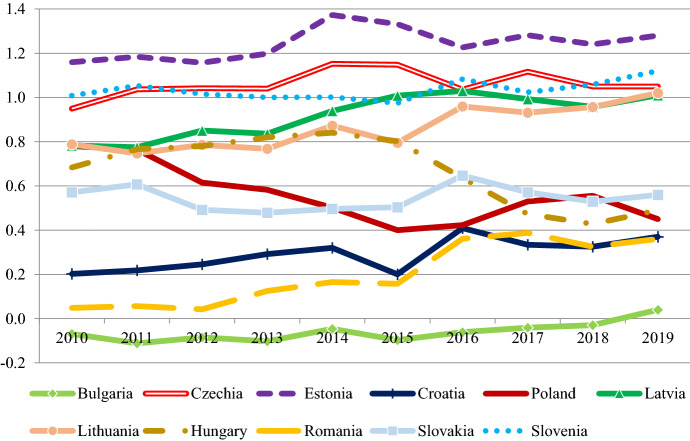
Rule of law index in the EU-11 (2010–2019) Notes: the figure shows the estimations for the World Governance Indicators rule of law index, which ranges between −2,5 and 2,5. Data: www.govindicators.org

Figure 3 provides an overview about the evolution of the rule of law in the EU-11 between 2010 and 2019 based on the World Bank's World Governance Indicators (WGI). The rule of law is conceptualized as capturing “perceptions of the extent to which agents have confidence in and abide by the rules of society, and in particular the quality of contract enforcement, property rights, the police, and the courts, as well as the likelihood of crime and violence”.^2^

There were three groups in 2010: the Southern countries (Bulgaria, Romania and Croatia) were lagging behind, Estonia, Czechia and Slovenia were at the forefront, while Latvia, Lithuania, Poland, Hungary and Slovakia were in the middle. Nearly a decade later, the midfield split in two, with Latvia and Lithuania catching up with the frontrunners, while Hungary, Poland and Slovakia deteriorating with Romania catching up with them. Bulgaria remains far behind in the region as its institutions have mostly stagnated at low levels over the decade.

**Fig. 4:**
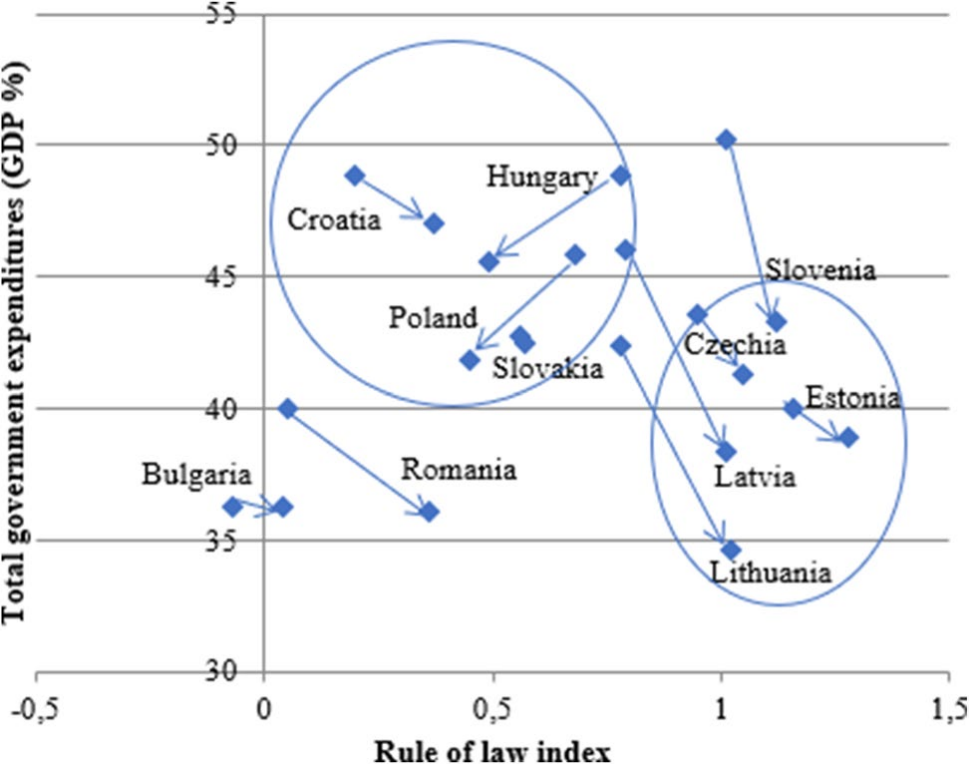
Changes in the rule of law index and total government redistribution (% GDP) in the EU-11 2010–2019. Note: the blue squares at the starting point of the arrows show the 2010 rule of law and total government expenditure combination, and the arrows end with the 2019 combination. Data: rule of law: see Fig. 3, total government expenditure rate: Eurostat (code TEC00023)

Considering the level of total government expenditure and rule of law together (Fig. 4) provides a clearer picture about the changing constraints on government power.

Total government expenditure decreased almost everywhere among the EU-11. Countries in the frontrunner group all improved their rule of law index, and they also succeeded in decreasing the redistribution rate—all of them are below 45% of GDP, and the three Baltic states are below 40%. While the size of the state decreased in Croatia, Hungary, Poland, and Slovakia as well, it is on average greater than in the first group. Together with their mostly deteriorating rule of law scores, we can conclude that the state is much less constrained in these countries than in the first group. Romania stands apart from both groups—it is converging to the second group in rule of law, while its low rate of state redistribution puts it closer to the frontrunners.

**Fig. 5:**
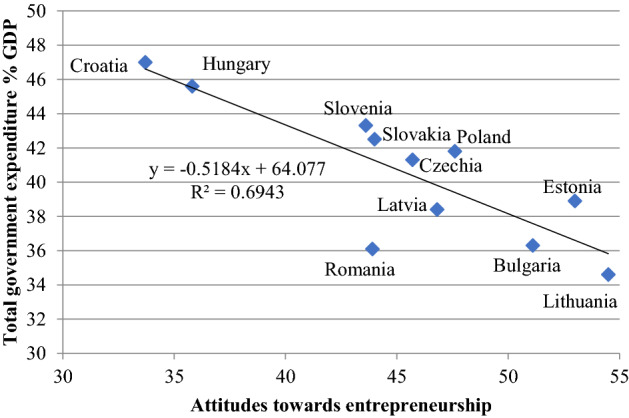
Total government expenditures and entrepreneurial attitudes in the EU-11 (2019). Data: World Economic Forum (2019) A*ttitudes towards entrepreunial risk* indicator in the country profiles; total government expenditures: see Fig. 4

On Fig. 5 we can see a strong correlation between entrepreneurial attitudes and the ratio of state redistribution—countries with a low redistribution rate have stronger entrepreneurial attitudes. Lithuania has the highest score, which has converged to the EU average the most dynamically in the last decade.

### Innovation and the quality of education

**Fig. 6:**
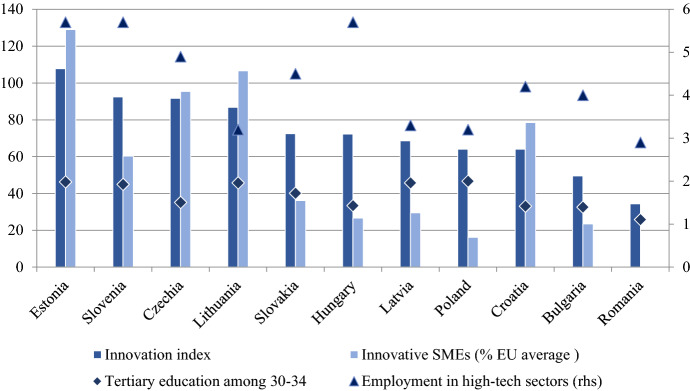
Selected indicators of innovation in the EU-11 (2019). Data: Innovation: European Commission, European Innovation Scoreboard interactive data analysis: https://interactivetool.eu/EIS/EIS_2.html#, Tertiary educational attainment among age group 30–34: Eurostat (code: T2020_41), Employment in high-tech manufacturing and knowledge-intersive services as % of total employment: Eurostat (code: htec_emp_nat2)

Between 2010 and 2020, there was no significant change in the fact that the EU-11 countries are less innovative than the old Member States. According to the European Innovation Scoreboard^3^ only Estonia belongs to the second group of Strong Innovators, which includes France and Germany. On the other end of the spectrum, we find Romania and Bulgaria as Modest Innovators, which perform below 50% of the EU average in almost all indicators. Other countries in the region are among the Moderate Innovators, performing between 50 and 100% of the EU average. From the perspective of MIT, the innovation performance of domestic small and medium-sized enterprises (SMEs) is of paramount importance. Figure 6 shows the innovation index of the EU-11 and the proportion of innovative SMEs. Except for Estonia and Lithuania, the share of innovative SMEs is below the EU average everywhere, with 6 countries (Slovakia, Hungary, Latvia, Poland, Bulgaria and Romania) below 40% of the EU average. The figure also shows the employment rate in high-tech manufacturing and services as well as the share of graduates in the 30–34 age group. As we can see there is only a moderate relationship with either the innovation index or the proportion of innovative SMEs. Hungary is one of the leaders in the high-tech employment indicator together with Estonia and Czech Republic even if it has a low share of innovative SMEs and a relatively low share of people with tertiary degree. This anomaly can be explained by the presence of foreign-owned companies in high-tech manufacturing sectors such as the auto industry. However, as Drahokoupil and Fabo (2020: 4) show, foreign-owned firms’ contribution to skill development is limited even for sophisticated products as assembly platform specialization requires little creative work from employees. Digital skills and creative work are required in domestic, cutting edge services sector, which underlines the significance of innovative SMEs.

**Fig. 7:**
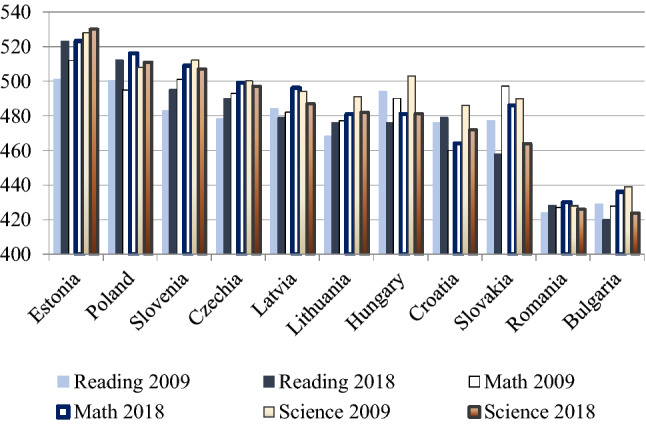
Results of the PISA tests in the EU-11 (2009 and 2018). Data: OECD (2009) and OECD (2019)

A high-quality education system is an essential condition for high-road competitiveness. Educational standards can be assessed through the PISA surveys carried out by OECD, which measures the performance of 15-year-old students in reading, mathematics, and science. Figure 7 shows the results of the 2009 and 2018 PISA tests for the EU-11.

The highest scores were registered by Estonia, which even increased its performance over the past decade and is one of the top performers in the world due to its strong focus on educational reforms (Avlijas 2020: 629). Poland also recorded steady improvement in all three areas. On the other hand, we can observe mostly stagnation at a low level in Romania and Bulgaria, while there is a general decline in all three areas in Slovakia and Hungary. In the remaining countries we can see both progress and decline.

### Wages and productivity

**Fig. 8:**
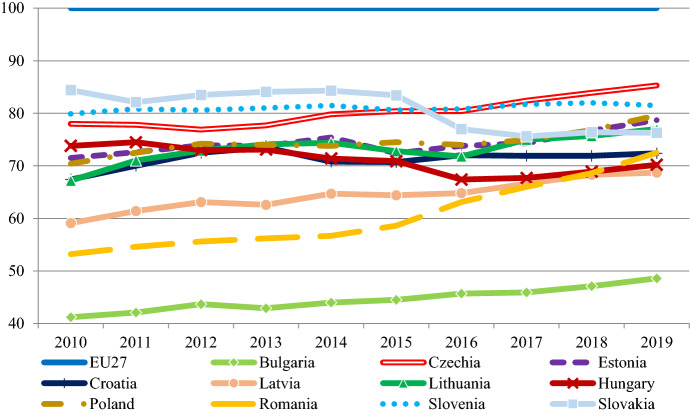
Labor productivity in the EU-11 (2010–2019). Notes: Labour productivity per person employed and hour worked (EU27 = 100) Eurostat code: TESEM160

The capacity for innovation and high-quality education contribute to productivity growth, which determines wage levels and economic demand in the longer term. Trends in productivity are shown by Fig. 8.

As we can see, the EU27 average has not yet been approached by any country of the region. The closest one is Czechia, which showed steady convergence over the past decade. The three Baltic States and Romania are also catching up rapidly. The latter has outpaced Hungary, which, together with Slovakia, has diverged from the EU27 average. Over the past decade, Poland was the only country, which registered dynamic productivity growth and has almost caught up with Slovenia despite its weakening institutional system and deteriorating rule of law.

**Fig. 9:**
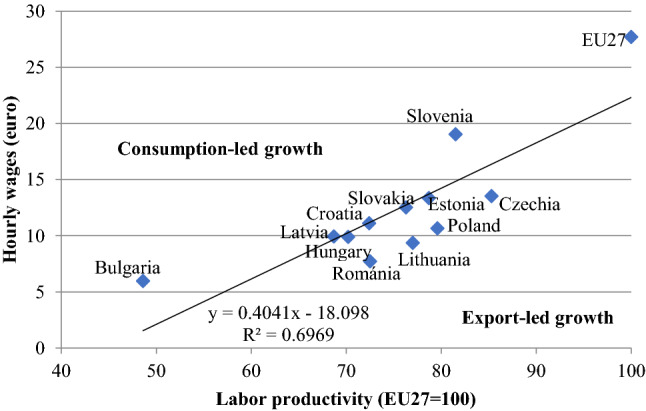
Hourly wages and labor productivity in the EU-11 (2019). Data: productivity see Fig. 8, wages: Eurostat (code: lc_lci_lev)

Figure 9 shows a strong link between labor productivity and hourly wages, but this relationship is not perfect. While Slovenia and Bulgaria are well above the trend line, indicating more consumption-led growth, most countries in the region, especially Czechia, Poland, Lithuania, and Romania are well below it, which implies an export-oriented growth path.

### The financial conditions of growth

**Fig. 10:**
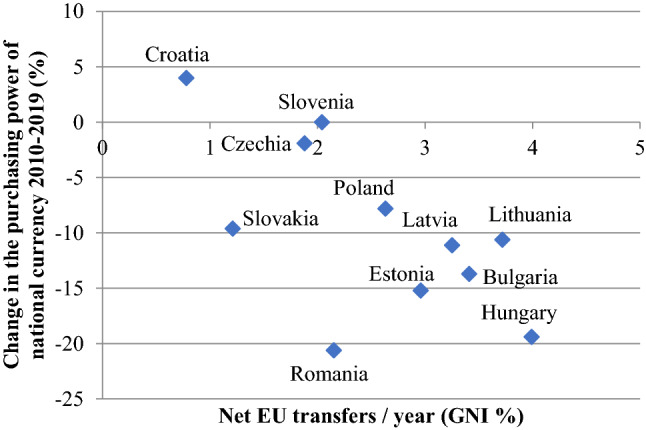
Net EU-transfers and change in the purchasing power of national currency in the EU-11 (2010–2019). Data: own calculation based on European Commission budget data: https://ec.europa.eu/budget/graphs/revenue_expediture.html Change in the purchasing power of national currency: own calculation based on Ameco GDP purchasing power parities, national currency units per purchasing power standard 2010/2019.

As discussed in the theoretical section, lax monetary conditions can also contribute to growth although long-term sustainability is highly questionable. A partial picture of the financial conditions for growth is provided by Fig. 10, which examines two indicators together: the level of net EU transfers as a percentage of gross national income (GNI) and the change in the value of the national currency, measured through AMECO's purchasing power standards (PPS). The figure shows that there were significant differences in the financial conditions for growth in the region. Slovenia and Czechia developed with stable money and EU transfers less than 2% of their respective GNI. The three Baltic countries received EU transfers between 3 and 4% of their GNI, while they also experienced a decrease in the purchasing power of money, which means rising prices even after introducing the euro. Bulgaria faced similar financial conditions. Croatia was the only country among the EU-11, where the purchasing power of the national currency increased over the past decade due to the euroization of its financial system.^4^ Poland and Slovakia are around average in terms of both EU transfers and exchange rate changes. The loosest monetary conditions were in Romania and Hungary—both registered around 20% reduction in the purchasing power of the currency and significant transfers from the EU—annually Romania received 2%, Hungary 3.99% as a proportion of GNI, the latter being the highest in the region.

Figure 10 tells only a partial story of monetary conditions. Beyond official transfers, countries in the region also received significant amount of remittances from those, who went to work abroad primarily to other EU countries. As emphasized by Csaba (2019: 10), the inflow in remittances is often comparable in magnitude to official transfers. In 2020 remittances as % of GDP ranged from 0,9% in Poland to 6,4% in Croatia with an average of 2,47% in the EU-11.^5^

Based on these numbers, we can see that the financial conditions of growth do not indicate a strong relationship with convergence performance. Loose monetary conditions can temporarily boost demand, but they do not necessarily contribute to improvement in productivity or competitiveness.

### Different growth models and the quality of life

Based on the previous sections, we can see that behind the steady convergence performances in the CEE region, three different paths can be observed. The most successful countries were those, where strong institutions, a focus on knowledge and favorable financial conditions have all contributed to growth. Czechia, Slovenia, Estonia, and Lithuania belong to this group. These countries have successfully resolved the problems presented by MIT and they have been able to pursue a growth model based on knowledge and quality. Convergence was possible for other countries as well, where institutions are weak or deteriorating. Romania and Hungary are the best examples. Their growth is based primarily on low costs and requires significant influx of financial resources as well as periodic devaluation. They are still in the MIT and this path is unlikely to be sustainable in the long-term. Countries, where there was little improvement in institutions or education, and financial conditions were less favorable, performed much weaker—Croatia, Bulgaria, Slovakia belong to this group.

However, growth is not the ultimate objective of economic activity. The issue of living standards is much more important for the population. This is expressed by the UN Human Development Index (HDI), which provides a combined assessment of life expectancy, education, and income.

Table 3 shows how it evolved between 2010 and 2020 in the EU-11 countries. In 9 of the EU-11 countries, HDI rankings improved in the 2010s—three of the region's best performing countries are already in the top 30 countries in the world (Slovenia, Czechia, Estonia). Poland, Lithuania, and Latvia also improved significantly and made the top 40. Slovakia is in this group as well, but it is also one of the two countries, which did not improve, and dropped from 31st place in 2010 to 39th place by 2020. Hungary also fell from 36 to 40^th^ place. Croatia is not far behind as it improved from 51 to 43th. The position of Romania and Bulgaria is largely stable.

**Table 3:** The ranking of EU-11 countries based on the Human Development Index (HDI) 2010, 2020

Country	2010	2020	Δ
Slovenia	29	22	+ 7
Czechia	28	27	+ 1
Estonia	34	29	+ 5
Lithuania	44	34	+ 12
Poland	41	35	+ 6
Latvia	48	37	+ 11
Slovakia	31	39	–8
Hungary	36	40	–4
Croatia	51	43	+ 8
Romania	50	49	+ 1
Bulgaria	58	56	+ 2

*Source*: UNDP (2010) and UNDP (2020)

Overall, a competitiveness strategy, which prioritizes institutional development and quality, is not only associated with better growth performance, but also with a better quality of life. Only Poland appears to be the exception in this regard, where, despite the deterioration of the rule of law indicator, there has been excellent performance both in terms of growth and HDI. This can be explained by the fact that between 1990 and 2015 successive governments built a system committed to free market and capitalism, which kept the country on a steady growth path. As Magyar and Madlovics (2020: 650–651) explain, even after 2015 the economy was mostly left intact and loyal members of the ruling party are rewarded by office and not wealth. It is a very different case from Hungary even with a similar rule of law deterioration.

The next section will look deeper into the Hungarian case as a representative case of deteriorating institutions and the persistence of the cost-based growth model. It will be contrasted with Estonia, which represents the high-road path to growth. The comparison of the two countries will help to understand the political processes, which lie behind the different growth models.

## Divergence in institutions and growth models in Estonia and Hungary

In 2010 Hungary and Estonia were roughly at the same level of development (Fig. 2). In the 1990s they were the frontrunners among post-Communist countries in committing to a strategy of FDI-led growth, which was followed by the rest of the region. From the beginning, their main difference can be found in the role of the state, which has clearly visible consequences in the developments of the post-2010 period. By analyzing these two cases, we can understand the relationship between the institutional system and the chances to overcome the MIT.

### Divergence in transition and institutions

A complete break with the Soviet past and accession to the West became an essential element of the emerging new Estonian identity following the collapse of the Soviet Union. In the economy, this was reflected in the adherence to a strict neoliberal policy, which includes the flat tax, a currency board exchange rate system, balanced budget, and rapid privatization to foreigners. The latter was primarily driven by fear so that the Russian minority would not acquire significant property (Bohle and Greskovits 2012: 100). The exclusion of Russian nationals from the political process significantly reduced the political obstacles to reforms. Following macroeconomic stabilization and trade liberalization, the new government, led by Mart Laar, focused on securing property rights and the rule of law. These were critical to attracting FDI, which was an essential element of the economic strategy. The reforms also included the fight against corruption, but there was no independent institution set up for this purpose—the solution was seen in radical market reforms, strengthening courts and civil society, and getting rid of people with ties to the Soviet past (Laar 2007: 7).

In contrast to Estonia, Hungary had a more consensual type of transition, during which there was no sharp break with the past. Even 30 years after the transition, the Kádár-era—named after the Communist leader of Hungary between 1956 and 1988—is still the most popular period in twentieth century Hungarian history, especially among those who were adults at the time (Szabó and Gerő 2019: 66). The economic transition began long before the first free elections, thus unlike in other countries of the region, a macroeconomic shock therapy, which means the parallel process of stabilization and liberalization, could be avoided, and a more gradual transition path followed (Csaba 1995: 195). Hungary was in fact at the forefront of the reforms due to its early decision to open privatization to foreign investors—given the significant level of inherited foreign debt, which needed to be financed (Bod 2019: 23–24). Continuity with the past was represented by the welfare state, the preservation of which was a social expectation, especially during the period of transformational recession and rising unemployment (Bohle and Greskovits 2012: 154). Paternalistic expectations and dependence on the state set the ground for profligate fiscal policies that led to the financial crisis in 2008 and weakened the potential for social resistance to authoritarian tendencies after 2010 (Győrffy 2020: 806).

**Fig. 11:**
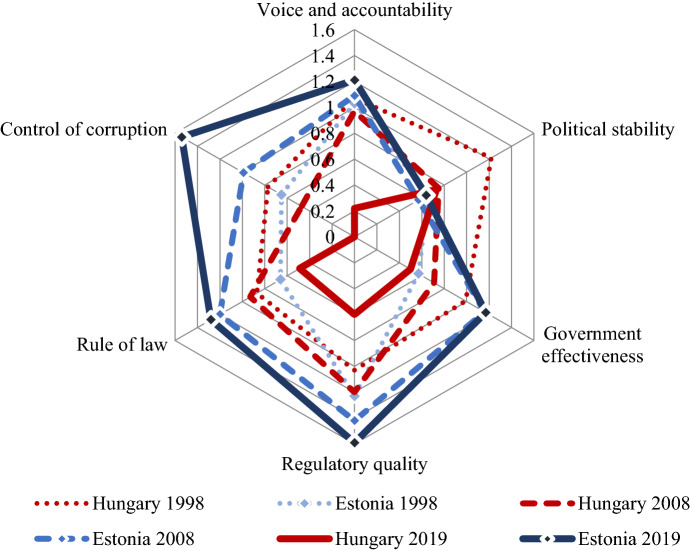
Quality of governance in Estonia and Hungary (1998, 2008, 2019). Data: World Governance Indicators, available: http://govindicators.org

The impact of different transitions could not be traced in the institutional quality of Estonia and Hungary in the early period of the transition. During the EU accession process, both countries implemented the necessary reforms that led to a stable improvement in the quality of governance. However, after accession, this process was reversed in Hungary, while it continued in Estonia. Figure 11 illustrates these trends based on the World Governance Indicators. The quality of governance is represented by the size of the area delineated by the different dimensions. As we can see on the figure, by 2018 an enormous gap took shape between the two countries.

In Estonia, the transition to a market economy also meant the spread of market solutions in public administration. The appointments were subject to competition and those, who held office during the Soviet system, had a significant disadvantage (Kalnins 2017: 112–113). The practices of new public management were introduced, which implied a wide-ranging decentralization—reflecting a keen distrust of all types of centralization due to the legacy of the Soviet past (Sarapuu and Saarniit 2020: 323).

The most visible element of institutional reforms in Estonia is the E-Estonia program, which serves transparency, efficiency, and the reduction of transaction costs in the economy. Since 2002, every citizen has a digital ID and signature that allows them to do almost everything online from paying taxes to voting—the exceptions are marriage, divorce and real estate sales. Vassil (2015: 19) estimates that even if we calculate with only 5 min saving of time in each interaction, the system has saved thousands of years by 2014. Presumably, this effect is also reflected in the strong entrepreneurial attitudes as shown in Fig. 4.

Hungary took a completely different path. After the accession to the EU, there was no longer any external incentive for the development of the institutional system. Once Viktor Orbán became prime minister with a constitutional majority in 2010, a completely new administrative strategy was formulated, which aimed at the centralization of public administration—a significant U-turn after decades of decentralization (Kornai 2015). The changes do not fit into public administration reform trends in the West but represent a new illiberal practice (Hajnal and Rosta 2019). The centralization of decision-making rights, the politicization of public administration, the devaluation of expertise and the weakening of traditional standards of bureaucracy are the main features (Hajnal 2021). These changes significantly weakened the institutional checks and balances providing the opportunity for the abuse of state power. This implies a business environment radically different from the Estonian case.

### Divergent approaches to growth

Estonian policymakers built the foundation for high-quality growth during the first decade of the transition. Since significant deindustrialization took place during the 1990s, they focused heavily on the services sector. Among the three most important services—transport, information and communication technology (ICT), construction—ICT was seen as the most promising. The knowledge base of the sector was the physical research institutes inherited from Soviet times, including the Cybernetics Institute, established in 1960, which proved to be a major asset for the new economy (Mets 2018: 91–92). Already in 1996, policymakers were paying attention to ICT, which appeared in the 1997 Tiger Leap ICT strategy—as a result 97% of schools connected to the Internet. In 2002, a program of knowledge-based Estonia was launched, using Michael Porter's model of competitive development as a reference. R&D expenditure was set to increase to 1.5% of GDP (Mets 2018: 93). An important part of the knowledge-based strategy was to improve the quality of the education system—already in 2002 Estonia spent 20% of the total government expenditure on education, while the same year Hungary spent only 11% (Avlijas 2020: 627). In addition, there is a strong emphasis on entrepreneurship education, which is taught at all universities (Mets 2017: 125). These factors explain the strong performance in various innovation indicators that was presented in the previous section.

Hungary opted for a very different path. After being a frontrunner in the transition with its FDI-led, export-based growth model, fiscal overspending from 2001 led to increasing debt levels, a significant current account deficit and eventually to the 2008 financial crisis.^6^ Following the crisis, macroeconomic policy focused on stabilizing imbalances and increasing employment (Matolcsy and Palotai 2019: 9). Unlike in Estonia, where the currency board and later the introduction of the euro in 2011 prevented the use of independent monetary policy, lower interest rates and the subsequent weakening of the Hungarian currency (Fig. 10) played a critical role in ensuring cost-based competitiveness of Hungarian products and balancing the current account. The employment rate was increased through cutting welfare payments and the deregulation of the labor market—the measures include the shortest unemployment benefit duration in the EU with 3 months, which is in sharp contrast with the 6–12 month duration in the Baltic states (Avlijas 2020: 635). Following the financial crisis, real unit labor costs (RULC) fell until 2015 and then grew only slowly—the wage share remained below 45% of GDP by 2019, compared with over 50% before the crisis.^7^ However, devaluation made the increase in wages in domestic currency possible, and together with the availability of cheap loans this contributed to the recovery in consumption, which also stimulated growth (Matolcsy and Palotai 2019: 16).

Beyond macroeconomic stabilization and increasing employment rate, the most important element of Hungarian economic policy is increasing the share of domestic ownership in selected sectors of the economy—to counter the dependence on FDI and strengthen the powerbase of the government. This was achieved through differential treatment of the manufacturing and the service sector. Mobile, manufacturing FDI is highly encouraged and supported—as a result, following the financial crisis, Hungary has been the most successful country in the region in attracting automotive FDI mainly due to low wages and significant state aid (Pavlinek 2017: 23). In contrast, the service sector became a target for nationalization, especially in energy, transport, and the financial sector (Voszka 2018: 1287–1288). New Hungarian owners were also created in the private sector—mainly through channeling EU funds to selected companies close to the government, especially in construction (Szanyi 2019: 15–16).

Macroeconomic consolidation and ownership restructuring in the economy did not require improving the institutional system—in fact, the dismantling of checks and balances was viewed as necessary in these processes.^8^ There was no incentive to invest in knowledge and education either—as shown by Fig. 6 and 7, Hungary fell behind in the share of graduates as well as PISA scores. Still, the government achieved its limited objectives—during the pre-coronavirus period macroeconomic imbalances disappeared, the employment rate increased, and Hungary registered a steady growth rate even if it is not outstanding by regional comparison. At the same time, the country has been unable to overcome the problem of MIT as productivity declined and even in the high-tech car industry there is a specialization on low-value added activities and a competitiveness based on low costs (Rechnitzer et al. 2017). Without institutional development, strengthening entrepreneurship and creating conditions for a knowledge-based economy, this strategy cannot lead to convergence.^9^ The Hungarian case thus stands in sharp contrast with Estonia, where high-quality institutions ensure forward looking policies and produce a favorable business environment, where entrepreneurs can thrive, and innovation can flourish.

## Conclusions

The paper provided an overview about the convergence performance of the CEE region between two major crisis—the 2009–2013 financial crisis and the COVID-19 pandemic. Two decades after the transition, the main task during this period was to overcome the MIT, which essentially means a shift from cost-based to quality-based growth. Except for Slovakia, all countries have moved closer to the EU average, but this quantitative similarity hides significant divergence—while the Czechia, Slovenia and Estonia are on track towards a knowledge-intensive, high-quality growth model, the rest of the region continues to compete primarily on a cost basis. Without increasing productivity, growth becomes a function of attracting additional labor and capital into the region, which has clear limits given the declining population and the expected decline of FDI-flows due to the new technological revolution.

The paper underlined the significance of institutions, especially the rule of law in making the transition from cost-based to quality-based growth. However, the comparative cases of Estonia and Hungary also indicated the problem of path dependency in institutional change—while a clean break with its Communist past helped Estonia in building a high-quality institutional environment, the lingering heritage of the Communist past in Hungary together with the financing opportunities provided by the EU contributed to institutional backsliding and the persistence of the cost-based growth model.

Neither the MIT, nor the problems presented by institutional path dependence are specific to the CEE region. Further research is needed to apply the findings to other regions with different heritage such as the Southern member states of the EU. Such analysis seems especially timely as the new budget of the EU for 2021–2027 and the Next Generation EU Fund provide the opportunity for EU member states to make the transition to a knowledge-intensive and high-quality growth model. Institutions are critical in this process given their impact on the business environment and the quality of policymaking. The inclusion of the rule of law conditionality is a significant step forward. Strictly enforcing the new regulations during the distribution of the funds can contribute to breaking path dependency and is probably as important for development as the funds themselves.

## Footnotes

1 The results depend strongly on the base year. 2010 was chosen because by then all the countries in the region had been through the biggest shock of the financial crisis and the recovery started (Fig. 1).

2 For a description of the rule of law indicator, see the World Governance Indicators website: www.govindicators.org.

3 The report and annual data are available at: https://ec.europa.eu/growth/industry/policy/innovation/scoreboards_en. Accessed: March 31, 2021.

4 Croatia has the highest level of euroisation in deposits and payments among non-eurozone EU countries (Dumičič, Ljubaj and Martinis 2018: 2), which limits the possibility for devaluation. Instead internal devaluation can be used to reduce costs, which means that wages grow at a lower rate than productivity. This is shown by the fact that while the purchasing power of the Croatian kuna increased, the real effective exchange rate (REER) decreased from 120,1 in 2010 to 98,8 by 2019 (2015 = 100). In contrast, the Hungarian forint was devalued by 25% nominally, but the REER only decreased from 110,3 to 103,4 during the same period (AMECO database). The devaluation of the forint made it possible for the government to implement popular wage increases without the loss of cost competitiveness.

5 Own calculation based on World Bank data available at: https://www.worldbank.org/en/topic/migrationremittancesdiasporaissues/brief/migration-remittances-data.

6 For an overview of the crisis see Győrffy (2018): 147–190 as well as Király (2020).

7 Data: AMECO database, code: ALCD0.

8 As a consequence, Hungary became a paradigmatic case of democratic backsliding. Discussing the specifics of this process is beyond the scope of this paper. For a comparative perspective of the transformation of the Hungarian political regime see f. ex. Győrffy (2020) or Magyar and Madlovics (2020).

9 This assessment is widely shared among Hungarian economists—see Csaba (2019).
